# Retentive capacity of power output and linear versus non-linear mapping of power loss in the isotonic muscular endurance test

**DOI:** 10.1038/s41598-021-02116-2

**Published:** 2021-11-22

**Authors:** Hong-qi Xu, Yong-tai Xue, Zi-jian Zhou, Koon Teck Koh, Xin Xu, Ji-peng Shi, Shou-wei Zhang, Xin Zhang, Jing Cai

**Affiliations:** 1 Research Center of Sports and Health Science, School of Sports Science and Physical Education, Northeast Normal University, Changchun, Jilin Province China; 2 Research Field of Medical Instruments and Bioinformation Processing, College of Instrumentation and Electrical Engineering, Jilin University, Changchun, Jilin Province China; 3 Physical Education and Sports Science Academic Group, National Institute of Education, Nanyang Technological University, Singapore, Singapore; 4 Research Field of Representation Learning On Graph and Date Mining, School of Information Science and Technology, Northeast Normal University, Changchun, Jilin Province China; 5 Ergonomics Standardization Research Field, China National Institute of Standardization, Beijing, China

**Keywords:** Fatigue, Muscle

## Abstract

The limit of dynamic endurance during repetitive contractions has been referred to as the point of muscle fatigue, which can be measured by mechanical and electrophysiological parameters combined with subjective estimates of load tolerance for revealing the human real-world capacity required to work continuously. In this study, an isotonic muscular endurance (IME) testing protocol under a psychophysiological fatigue criterion was developed for measuring the retentive capacity of the power output of lower limb muscles. Additionally, to guide the development of electrophysiological evaluation methods, linear and non-linear techniques for creating surface electromyography (sEMG) models were compared in terms of their ability to estimate muscle fatigue. Forty healthy college-aged males performed three trials of an isometric peak torque test and one trial of an IME test for the plantar flexors and knee and hip extensors. Meanwhile, sEMG activity was recorded from the medial gastrocnemius, lateral gastrocnemius, vastus medialis, rectus femoris, vastus lateralis, gluteus maximus, and biceps femoris of the right leg muscles. Linear techniques (amplitude-based parameters, spectral parameters, and instantaneous frequency parameters) and non-linear techniques (a multi-layer perception neural network) were used to predict the time-dependent power output during dynamic contractions. Two mechanical manifestations of muscle fatigue were observed in the IME tests, including power output reduction between the beginning and end of the test and time-dependent progressive power loss. Compared with linear mapping (linear regression) alone or a combination of sEMG variables, non-linear mapping of power loss during dynamic contractions showed significantly higher signal-to-noise ratios and correlation coefficients between the actual and estimated power output. Muscular endurance required in real-world activities can be measured by considering the amount of work produced or the activity duration via the recommended IME testing protocol under a psychophysiological termination criterion. Non-linear mapping techniques provide more powerful mapping of power loss compared with linear mapping in the IME testing protocol.

## Introduction

Most human activities such as sports, industrial tasks, and daily living activities require dynamic endurance because most muscles are required to work continuously to accomplish these activities^1–3^. The limit of human endurance during a physical task is usually decided by the point at which an individual is unwilling or unable to continue the physical task, and this point is referred to as the point of ‘exhaustion’ or ‘fatigue’^4,5^. Muscular endurance is thus the measurement of an individual’s level of stamina or fatigue, and it may be limited by numerous physiological, biomechanical, mechanical, and psychological factors^6–8^. Many investigators have defined neuromuscular fatigue as a decreased performance under certain conditions^9,10^. During dynamic movement, neuromuscular fatigue can be directly assessed by any exercise-induced reduction in the ability to generate power or quantified as a time-dependent loss in power output^11–14^. Furthermore, recording surface electromyography (sEMG) during activities is useful to assess and understand muscle fatigue or endurance involved in muscle excitation, recruitment, and contraction^12,15–17^. Hence, sEMG is commonly used as an indirect and non-invasive method for assessing neuromuscular fatigue alongside mechanical variables (e.g., losses of force, torque, or power output)^12,18^. Furthermore, psychological fatigue is often incorporated as a part of the neuromuscular model of fatigue because fatigue is a psychophysiological symptom underpinned by interactions between performance fatigability and perceived fatigability^8,19,20^. Psychological alterations and perceived exertion determine the unconscious perception of fatigue, which can reduce power output and lead to the point of exhaustion^6,8,21^.

Specifically, power output, a combination of dynamic torque and joint angular velocity, and the duration of repetitive contractions are the two key contributors to dynamic endurance^22–24^. Thus, the retentive capacity of muscular power output is of great importance to most kinds of movements^2,14^. In previous studies, the isometric, isokinetic, or isotonic modes on dynamometers have been used to test muscular power^2,3,25,26^. Isotonic testing is considered to be more relevant to normal voluntary contractions, as the load is held constant but the velocity can vary^27,28^, which allows for the measurement of normal human movements during both acceleration and deceleration without controlling the angular velocity and acceleration^25,27–29^. Therefore, the isotonic testing protocol and database of muscular endurance is more applicable to the evaluation of sports science, rehabilitation medicine, and human-factor engineering. Moreover, the relationship between sEMG and power loss is useful for assessing and understanding neuromuscular fatigue, as it can be defined on the basis of electrophysiological or mechanical events^30–33^, and linear techniques are often used to estimate muscle fatigue by relating changes in sEMG parameters to changes in power loss. However, myoelectric signals can be better modeled as outputs of a non-linear dynamic system rather than as random stochastic signals, so a non-linear model based on a learning procedure would provide more accurate tracking of power loss using sEMG variables compared to a linear model^12,31,34,35^. Additionally, combining the subjective estimates of load tolerance and the subjects’ determinations of their own muscular endurance is beneficial for revealing the subjects’ real-world capacity during muscular endurance experiments^20,36^, and the results can help balance the workload and physical capacity to prevent acute or overuse injuries in actual application^37,38^. However, in many previous studies, subjects could not independently determine their load tolerance in dynamic fatiguing exercises, which involved fixed sets, repetitions, and rest times with the individual maximum load (e.g., repetition maximum, RM)^15,30–33,39^, so the actual muscle fatigue experienced by the subjects in those exercises may have exceeded their voluntary limits.

There is a limited amount of information regarding neuromuscular fatigue assessed during isotonic loading protocols. However, such protocols are the most suitable for measuring dynamic muscular endurance during the everyday movements of humans because these movements can be characterized by ballistic sinusoidal changes in velocity with constant loads rather than movements of constant velocity^2,3^. Therefore, in this study, the retentive capacity of muscular power output was assessed for the plantar flexors as well as the knee and hip extensors of college-aged males using an isotonic dynamometer and considering the subjective estimates of muscle fatigue. The isotonic endurance data of the lower limb muscles accumulated from the tests provide further insights into the relationships between human performance and muscle endurance required in real-world activities^19,40^. In addition, to guide the development of more effective electrophysiological evaluation methods of muscular fatigue, the accuracy of a fatigue linear model and a non-linear model were compared for their ability to relate sEMG variables to power loss during dynamic contractions.

## Methods

### Subjects

Subjects were recruited through advertisement and invitations to participate in the study in a non-random manner. The study was approved by the medical ethics committee of Jilin University and was performed in accordance with relevant guidelines and regulations of the institutional review board after each subject had given written informed consent, and all procedures performed in studies involving human participants were in accordance with the Declaration of Helsinki. Forty healthy college-aged males (age, 21.22 ± 1.10 years; height, 173.81 ± 3.44 cm; weight, 65.29 ± 6.49 kg; body mass index, 21.61 ± 2.09 kg/m^2^) with no previous history of lower extremity or severe musculoskeletal injury participated in the study.

### Experimental procedure

A BTE Primus RS dynamometer (Dynatracä, BTE Co., Hanover, MD, USA) and Noraxon surface electromyography system (Noraxon, Inc. Scottsdale, AZ, USA) were used to collect data synchronously during the tests. Each subject performed a specific warm-up for the lower limbs and was familiarized with the experimental setup before the testing procedures. Next, each subject’s skin was dry-shaved and cleaned with alcohol. Then sEMG activity during the contractions of the right leg muscles was recorded from the medial gastrocnemius (MG), lateral gastrocnemius (LG), vastus medialis (VM), rectus femoris (RF), vastus lateralis (VL), gluteus maximus (GM), and biceps femoris (BF) by a pair of bipolar surface electrodes. Because the interelectrode distance influences the pick-up area, crosstalk, and signal spectrum^41–44^, the commonly used 22 mm spacing was selected for the interelectrode distance to enable quantitative comparisons of measured values with previous studies^30–32^.

**Figure 1:**
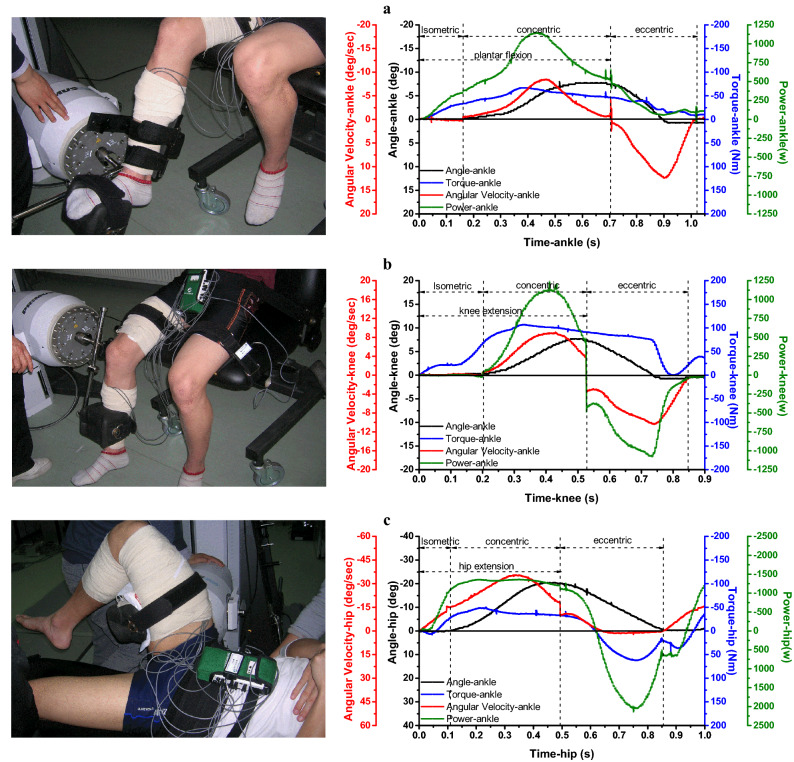
The change of angle, angular velocity, torque and power output with time during each cycle of the IME test for plantar flexors and knee and hip extensors. *Note* IME, isotonic muscular endurance. Each cycle included a limb loop in which a limb moves from the starting position to the ending position and then returns to the starting position. The angular presented a bell-curve, indicating that the limb loop moves from the starting position to the ending position, and then returns to the starting position. Correspondingly, with the two acceleration and deceleration movements of the limb segment, and the angular velocity presents two bell-shaped curves with opposite directions.

The protocol consisted of three sets of isometric peak torque (IPT) tests and one set of an isotonic muscular endurance (IME) test for the plantar flexors and the knee and hip extensors (Fig. 1). The right ankle, knee, and hip of the subjects were aligned with the mechanical axis of rotation of the dynamometer in a reclining position, sitting position, and lying position. For the IPT test, three repetitions of maximum voluntary isometric contraction (MVC) were required, where each contraction lasted for 3 s, and there was a 5 s rest period between contractions. A coefficient of variation (CV) of less than 10% was required for the three contractions. Each subject performed gradually increasing isometric contractions with the ankle plantar flexed at 80°, knee extended at 110°, and hip extended at 90°. For the IME test, the resistance level was set to 50% of the subject’s average IPT, and the subject performed a complete repetition for each cycle according to a timing cycle (45–60 cycles/min) displayed on the screen. Specifically, the timing cycle asked subjects to match the pace of a red horizontal “pacing bar” and to keep the work rate the same for the duration of the test. The subjects were asked to keep their range of motion and pace of each cycle consistent and continued the repetitions until they were fatigued. Each subject performed repetitive concentric contractions in the ranges of 80°–130° for ankle plantar flexion, 80°–170° for knee extension, and 90°–180° for hip extension. Although a subject’s fatigue point was indicated by the dynamometer when the amount of power output recorded in two consecutive cycles was below 75% of the amount of power output generated during the first 5 s, verbal encouragement was also provided to motivate the subjects to continue with the test until they experienced fatigue and decided to stop.

### Data acquisition and signal analysis

The length of lever arm (cm), force (N), and torque (N∙m) in three trials of the IPT test, and the work (J), time (s), and distance (°) in one trial of the IME test were recorded by BTE Primus^RS^ software. Both the sEMG signals and biomechanical signals such as time (s), torque (N∙m), angular position (°), and angular velocity (°/s) of the BTE Primus^RS^ dynamometer were recorded simultaneously by the TeleMyo 2400 T system (Noraxon, Inc., Scottsdale, AZ, USA) at a sampling rate of 1.5 kHz. Data analysis was performed offline using MATLAB 2014a software (MathWorks Inc., Natick, MA, USA).

Before acquiring the sEMG signal, an analog low-pass filter was used to suppress the high-frequency noise greater than 750 Hz in the system to eliminate the effect of its aliasing on the signal restoration. Then, a band-pass filter composed of a 5th-order high-pass filter and a 10th-order low-pass filter was adopted to digitally filter the collected sEMG signal to retain the frequency range of the signal within 20–250 Hz. Since wavelet transforms can realize the filtering function, wavelet transforms and the Hilbert transform were used when calculating IMNF and IMDF, and the cut-off frequency was 11.7188–350 Hz. In addition, the data of torque, angular position, angular velocity, and power output were smoothed by the adjacent averaging method. The angle of the limb segment increased from zero to the peak value or the positive bell-shaped part of the angular velocity, which was the concentric contractions phase of plantar flexors and knee and hip extensors (Fig. 1). The peak power output and sEMG parameters discussed in “Linear techniques used to estimate muscle fatigue”–“Time-dependent power output and power loss prediction” sections were calculated.

#### Linear techniques used to estimate muscle fatigue

##### Amplitude-based parameters and spectral parameters

Two main sEMG amplitude-based parameters, mean absolute value (MAV) and root mean square (RMS), were used to quantify the amplitude or magnitude of the sEMG signal^12,28,45^. Mean frequency (MNF) and median frequency (MDF) were estimated from the power spectrum using Fourier transform (FT). These two spectral parameters were used to quantify the changes in the spectral content of the sEMG signal^12,13,17,30,45,46^.

##### Instantaneous frequency parameters

The sEMG data were analyzed offline with a continuous wavelet transform (CWT) using MATLAB software with the signal processing and wavelet toolbox^32,47^. The Harr wavelet (db1 wavelet) was used as the mother wavelet, and the sEMG data were decomposed into six layers by the wavelet transform. The approximate scale of wavelet coefficients was retained, and the wavelet coefficients on the high-frequency details were removed to eliminate the clutter. The reserved wavelet coefficients were used to reconstruct the sEMG signal and complete the denoising process after the wavelet transform; the instantaneous mean and median frequency (IMNF and IMDF) of sEMG data were calculated by the Hilbert transform (HT)^13,17,48,49^.

#### Non-linear techniques used to estimate muscle fatigue

A multi-layer perception neural network (MLPNN) was chosen to relate changes in sEMG variables and power output because it shows good accuracy to relate sEMG variables and fatigue indices^12,31,35^. Four sEMG parameters were calculated from each contraction of all of the subjects: (1) MAV is an estimate of the mean absolute value of the signal, as the integrated EMG is divided by the integration time^15,34,50^; (2) zero crossing (ZC), as the number of times that the waveform crosses zero, is a simple measure of the main frequency of the signal^15,34,50^; (3) slope sign change (SSC), as the number of times that the slope of the waveform changes sign, provides another measure of frequency content^15,34,50^; and (4) wavelength (WL) provides information on the waveform complexity in each segment^15,34,50^. These parameters contain both amplitude and frequency information and have been shown to be useful in other sEMG pattern recognition applications^15,31,51^. The sEMG data were analyzed offline with MLPNN using MATLAB software with the signal processing and neural network toolbox. The training function adopted Levenberg–Marquardt backpropagation, which includes the gradient descent Weight algorithm and bias items, to learn the parameters of MLPNN. The number of hidden layer nodes was 10, the maximum number of training iterations was set to 100, the maximum mean square deviation of training results was set to 0.00004, the learning rate was set to 0.1, and the maximum number of failures was set to 18.0. Peak power output and sEMG parameters of all the subjects were subdivided into four training segments and one validation segment. This process resulted in a set of 4 × 1 training vectors and validation vectors for each subject and each test condition. Finally, the actual value (AV) and estimated value (EV) of the peak power output in the training-prediction process of five groups were compared to evaluate the performance of MLPNN.

#### Time-dependent power output and power loss prediction

The sEMG variables (individually and in combination) were used to estimate the power loss, and the relationship between sEMG variables and peak power output of all the subjects was mapped by the artificial neural network or linear regressions. The performance of different approaches was quantified by the signal-to-noise ratio (SNR) for the outputs^2,15,31,32,51^.

### Statistical analysis

The results of torque in the IPT test, and the number of repetitions, work, time, and distance in the IME test were compared using analysis of variance (ANOVA) and multiple comparisons. The power outputs (W) of the first five repetitions and last five repetitions were compared using paired-samples *t*-test. The association between changes in the percentage of power output and changes in the percentage of sEMG-based parameters, and the relationship between the estimated changes and actual changes in power output of MLPNN were determined by Pearson’s correlation coefficients (*r*) and multiple linear regression. The differences of slope and intercept from two linear regressions were determined by *F*-test or equivalent *t*-test. *P*-values less than 0.05 were considered statistically significant (two-tailed).

### Ethics approval

The study was approved by the medical ethics committee of Jilin University and was performed in accordance with relevant guidelines and regulations of the institutional review board after each subject had given written informed consent, and all procedures performed in studies involving human participants were in accordance with the Declaration of Helsinki.

### Consent to participate

Each participant provided informed written consent prior to participation.

### Consent for publication

Additional informed consent was obtained from all individual participants for whom identifying information is included in this article.

## Results

### The results of IPT and IME tests for plantar flexors and knee and hip extensors

**Figure 2:**
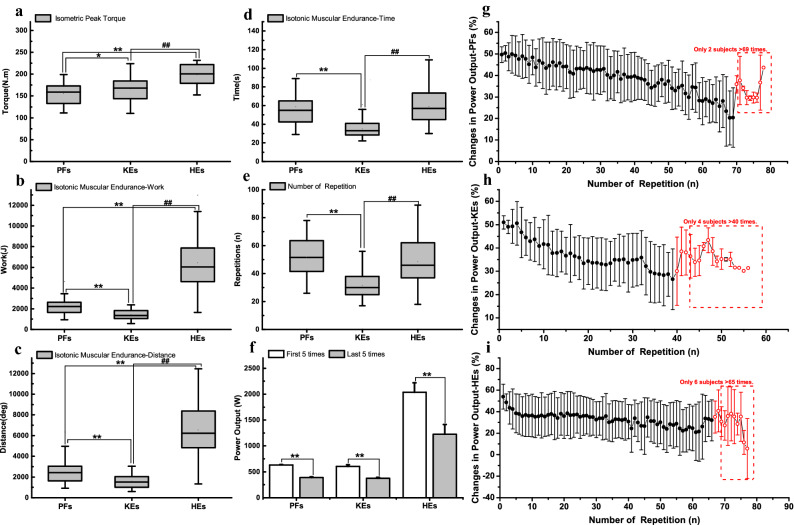
The result of IPT and IME test for plantar flexors and knee and hip extensors. *Note*: *IPT* isometric peak torque, *IME* isotonic muscular endurance. *PFs* plantar flexors, *KEs* knee extensors, *HEs* hip extensors. The comparison between PFs and KEs, PFs and HEs, or the power output of the first 5 repetitions and last 5 repetitions, * is *p* < 0.05, * * is *p* < 0.01; the comparison between KEs and HEs, ^#^ is *p* < 0.05, ^##^ is *p* < 0.01.

The IPT test was first performed to determine the appropriate resistance level for the IME test (i.e., 50% IPT). The results of torque for the plantar flexors and knee and hip extensors were 154.90 ± 25.17 N∙m, 166.45 ± 27.53 N∙m, and 198.56 ± 24.53 N∙m in the IPT test, respectively. Distinct increments were evident in the three muscle groups (*P* < 0.05) (Fig. 2a). Furthermore, the values of CV were all less than 10%, and they were 5.13 ± 3.10%, 5.11 ± 2.63%, and 4.46 ± 3.21% for the plantar flexors and knee and hip extensors, respectively.

The values of work, distance, and time in the IME test from low to high were the knee extensors (1407.28 ± 456.93 J, 1582.90 ± 635.88°, and 35.20 ± 8.77 s), plantar flexors (2259.56 ± 771.88 J, 2431.07 ± 1088.14°, and 58.65 ± 25.43 s), and hip extensors (6460.69 ± 2521.18 J, 6550.79 ± 2421.75°, and 59.10 ± 18.13 s). Distinct increments were evident in these muscle groups for the work and distance (*P* < 0.01) (Fig. 2b–d). The number of repetitions in the IME test from low to high were the knee extensors (31.48 ± 9.52), hip extensors (48.42 ± 16.51), and plantar flexors (51.84 ± 13.30). Regarding the number of repetitions and time in the IME test, the results of the hip extensors and plantar flexors were significantly higher than the results of the knee extensors (*P* < 0.01) (Fig. 2d, e).

Two subjects repeated ankle plantar flexion more than 69 times, four subjects repeated knee extension more than 40 times, and six subjects repeated hip extension more than 65 times (Fig. 2g–i). Except for these more powerful subjects, the power output measured from all other subjects decreased continuously with the number of repetitions for plantar flexors and knee and hip extensors. The power output had a significant difference between the first five repetitions and the last five repetitions for plantar flexors (629.62 ± 13.27 W vs. 388.73 ± 15.96 W), knee extensors (606.21 ± 30.20 W vs. 377.47 ± 15.30 W), and hip extensors (2036.58 ± 180.96 W vs. 1227.73 ± 186.19 W) (*P* < 0.01) (Fig. 2f).

### The association between changes in percentage of power output and changes in percentage of sEMG-based parameters

**Figure 3:**
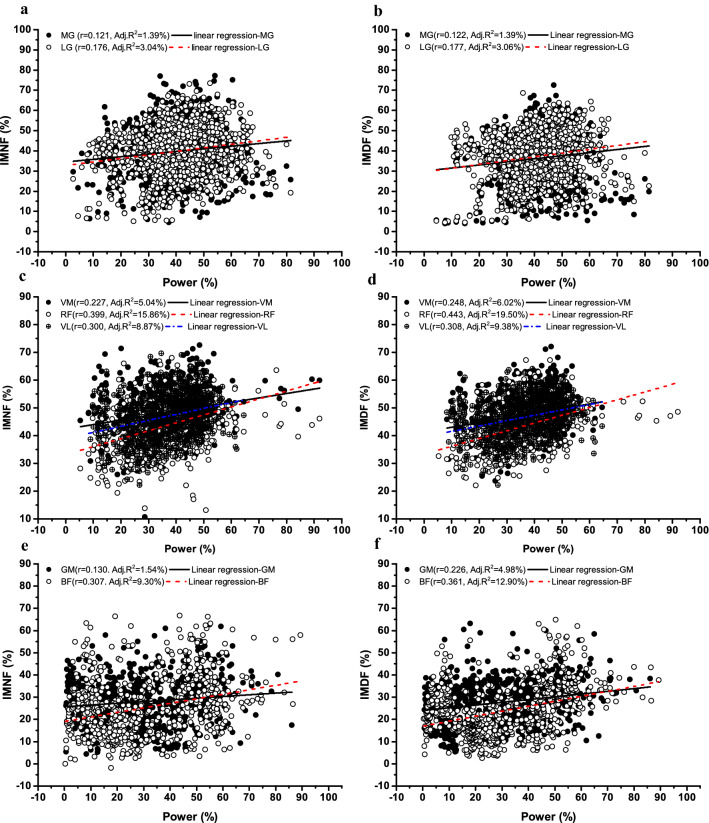
The association between changes in percentage of power output and changes in percentage of instantaneous frequency parameters (percentage of the first two repetitions). *Note*: *IMNF* instantaneous mean frequency, *IMDF* instantaneous median frequency. *MG* medial gastrocnemius, *LG* lateral gastrocnemius, *VM* vastus medialis, *RF* rectus femoris, *VL,*vastus lateralis, *GM* gluteus maximus, *BF* biceps femoris.

As shown in Table 1 and Fig. 3, the changes of power output were inversely correlated with amplitude-based parameters, the correlations were significant in most muscle groups (*r* = 0.077–0.215, *P* < 0.05), and RMS percentage (RMS%) had a higher correlation coefficient than MAV percentage (MAV%). Also, the changes of power output were positively correlated with spectral parameters, the correlations were significant in most muscle groups (*r* = 0.078–0.301, *P* < 0.05), and MDF percentage (MDF%) had a higher correlation coefficient than MNF percentage (MNF%). Moreover, the changes of power output were also positively correlated with instantaneous frequency, the correlations were significant in all muscle groups (*r* = 0.121–0.443, *P* < 0.01), and IMDF percentage (IMDF%) had a higher correlation coefficient than IMNF percentage (IMNF%). Furthermore, all of the correlation coefficients and most of the SNRs of instantaneous frequency (*SNR* = 4.811–8.341) were higher than amplitude-based parameters (*SNR* = 4.438–7.763) and spectral parameters (*SNR* = 3.971–7.878).

**Table 1:** Percent changes of linear time–frequency parameters against percent changes of peak power output and signal-to-noise ratio.

Muscle groups			Amplitude-based parameters		Spectral parameters		Instantaneous frequency	
			RMS%	MAV%	MNF%	MDF%	IMNF%	IMDF%
PFs	MG	r (R^2^)	− 0.119**(1.42)	− 0.117**(1.37)	0.078**(0.61)	0.104**(1.08)	0.121**(1.46)	0.122**(1.49)
		SNR	6.264	6.781	6.359	6.362	7.453	7.926
	LG	r (R^2^)	− 0.094(0.88)	− 0.085*(0.72)	0.148**(2.19)	0.170**(2.89)	0.176**(3.10)	0.177**(3.13)
		SNR	5.429	6.096	6.274	7.184	7.174	7.099
KEs	VM	r (R^2^)	− 0.215**(4.62)	− 0.167**(2.79)	0.093*(0.86)	0.123**(1.51)	0.227**(5.15)	0.248**(6.15)
		SNR	7.763	7.418	5.671	5.235	7.715	8.301
	RF	r (R^2^)	− 0.159**(2.53)	− 0.117**(1.37)	0.147**(2.16)	0.233**(5.43)	0.399**(15.92)	0.443**(19.62)
		SNR	7.334	7.208	7.878	7.724	8.136	8.341
	VL	r (R^2^)	− 0.160**(2.56)	− 0.135**(1.82)	0.054(0.29)	0.121**(1.46)	0.300**(9.00)	0.308**(9.49)
		SNR	7.586	7.558	7.593	7.374	7.726	7.727
HEs	GM	r (R^2^)	− 0.070(0.49)	− 0.069(0.48)	0.088*(0.77)	0.101*(1.02)	0.130**(1.69)	0.226**(5.11)
		SNR	4.438	4.438	4.096	3.991	4.811	4.974
	BF	r (R^2^)	− 0.101**(1.02)	− 0.077**(0.59)	0.244**(5.95)	0.301**(9.06)	0.307**(9.42)	0.361**(13.03)
		SNR	4.784	4.773	3.971	5.012	4.843	5.115

*PFs* plantar flexors, *KEs* knee extensors, *HEs* hip extensors. *MG* medial gastrocnemius, *LG* lateral gastrocnemius, *VM* vastus medialis, *RF* rectus femoris, *VL* vastus lateralis, *GM* gluteus maximus, *BF* biceps femoris. *r* pearson's correlation coefficients, *R*^2^ coefficient of determination, *SNR* signal−to−noise ratio. *RMS* root mean square, *MAV* mean absolute value, *MNF* mean frequency, *MDF* median frequency, *IMNF* instantaneous mean frequency, *IMDF* instantaneous median frequency.

*P < 0.05, **P < 0.01, the correlations were significant.

### The difference between linear regression and non-linear neural network for predicting the power loss

**Figure 4:**
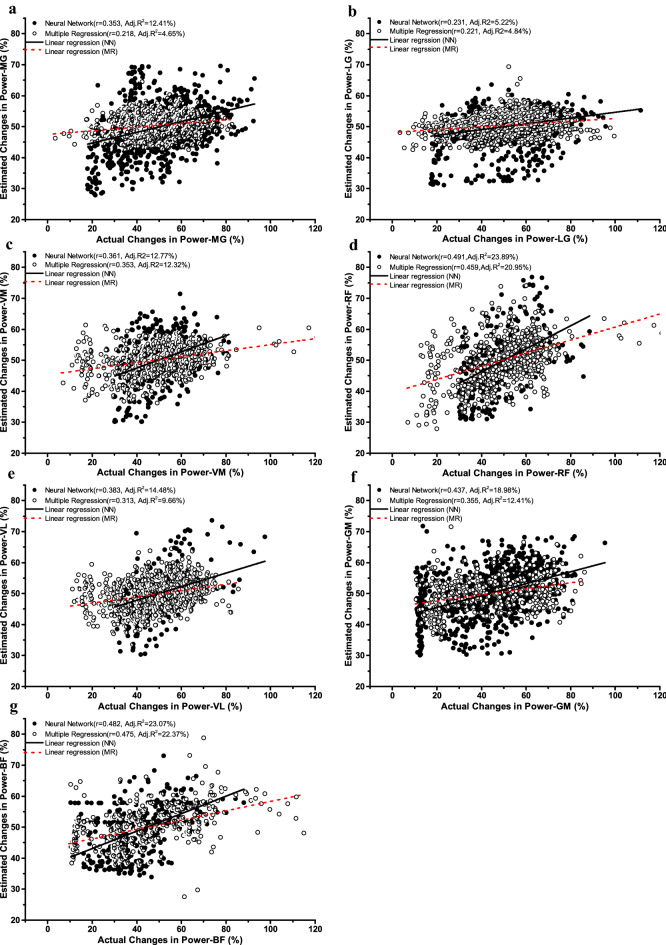
Actual changes versus estimated changes in peak power output obtained from both linear and non-linear models. *Note*: *r* pearson's correlation coefficients, *R*^2^ coefficient of determination. *NN* neural network, *MR* multiple regression. *MG* medial gastrocnemius, *LG* lateral gastrocnemius, *VM* vastus medialis, *RF* rectus femoris, *VL* vastus lateralis, *GM* gluteus maximus, *BF* biceps femoris.

The square of Pearson’s correlation coefficient (*r*) was equal to the coefficient of determination (*R*^2^) of univariate linear regression, where *R*^2^ reflects the percentage of total variation of a dependent variable that can be explained by the regression relationship of an independent variable. Thus, RMS%, MAV%, MNF%, MDF%, IMNF%, and IMDF% as single parameter predictors accounted for 1.02–4.62%, 0.59–2.79%, 0.61–5.95%, 1.02–9.06%, 1.46–15.92%, and 1.49–19.62% of the performance variance of changes in power output, respectively (Table 1 and Fig. 3). Stepwise multiple linear regression showed that the combination of sEMG-based parameters provided more accurate power mapping in a linear mapping than a single sEMG-based parameter predictor and accounted for 5.52–23.72% of the performance variance of changes in power output (Table 2). Moreover, the non-linear neural network based on the combination of sEMG-based parameters also provided higher correlation coefficients and SNR values (*r* = 0.231–0.491; *SNR* = 6.692–11.652) compared to multiple linear regression (*r* = 0.218–0.475; *SNR* = 5.555–8.590). Furthermore, the comparison between the regression lines of actual versus estimated changes in power output for both techniques indicated statistical significance (*P* < 0.01) (Table 2 and Fig. 4).

**Table 2:** The difference between linear regression and non-linear neural network for predicting the power loss.

		Linear regression of sEMG-based parameters			Actual versus predicted	Non-linear neural network		Linear versus non-linear			
								Slope		Intercept	
Muscle groups		R^2^	Regression equation	SNR	r	r	SNR	*F*	*P*	*F*	*P*
PFs	MG	11.02	Power% = 0.176 × IMNF% + 0.064 × MNF% + 32.473	8.590	0.218	0.353	9.040	38.89	0.000	0.08	0.780
	LG	5.52	Power% = 0.134 × IMNF% + 0.068 × MDF% + 33.891	7.199	0.221	0.231	8.720	15.30	0.000	0.01	0.911
KEs	VM	8.24	Power% = 0.340 × IMNF% − 0.151 × MAV% + 29.999	7.668	0.353	0.361	8.244	30.54	0.000	0.05	0.816
	RF	21.07	Power% = 0.679 × IMDF% + 0.073 × MDF% − 0.212 × MAV% + 15.220	7.669	0.459	0.491	8.916	19.18	0.000	0.00	0.957
	VL	9.67	Power% = 0.421 × IMNF% − 0.050 × RMS% + 20.875	8.527	0.313	0.383	10.103	21.60	0.000	0.00	0.959
HEs	GM	13.76	Power% = 0.626 × IMDF% + 9.881	5.555	0.355	0.437	6.692	23.20	0.000	0.00	0.996
	BF	23.72	Power% = 0.290 × IMDF% + 0.414 × MDF% + 5.833	5.761	0.475	0.482	11.652	23.21	0.000	0.59	0.441

*PFs* plantar flexors, *KEs* knee extensors, *HEs* hip extensors. *MG* medial gastrocnemius, *LG* lateral gastrocnemius, *VM* vastus medialis, *RF* rectus femoris, *VL* vastus lateralis, *GM* gluteus maximus, *BF* biceps femoris. *r* pearson's correlation coefficients, *R*^2^ coefficient of determination, *SNR* signal-to-noise ratio. *RMS* root mean square, *MAV* mean absolute value, *MNF* mean frequency, *MDF* median frequency, *IMNF* instantaneous mean frequency, *IMDF* instantaneous median frequency. Changes in percentage of power output (power%) was the dependent variable and changes in percentage of sEMG-based parameters such as RMS%, MAV%, MNF%, MDF%, IMNF%, and IMDF% were the predictor variables in these stepwise multiple regressions.

## Discussion

Losses of force, torque, and power output are typically used as direct mechanical variables to measure muscle fatigue^11–14^. Historically, muscle fatigue has been defined as the inability to maintain the required or expected force in isometric testing protocols. This determination of muscular endurance emphasizes the maximum force capacity and assumes strong muscles are also more powerful muscles^2,3,52^. In the present study, IPT was measured first to determine the appropriate resistance level for the IME test. As a larger muscle has a stronger force-generating capability with a greater physiologic cross-sectional area^22,23,53^, IPT exhibited a distinct increment in the plantar flexors, knee extensors, and hip extensors. However, isometric testing can only test the ability of the neuromuscular system to generate force or torque rapidly, and it does not determine muscular power, as its stationary nature prevents any assessment of velocity^53,54^. Therefore, the dynamic assessment of fatigue is not interchangeable with the isometric assessment of fatigue^52^, and optimal performance of dynamic contractions requires not only muscle force production but also a velocity component^2,24^. The order of IME differed from the order of IPT for the three tested muscle groups, showing that the ability to sustain maximal power output has a different physiological basis than muscle strength^52^. Specifically, knee extensors had the weakest endurance, with moderate torque output and minimum repetitions, plantar flexors had moderate endurance with minimum torque output and maximum repetitions, and hip extensors had the most powerful endurance with maximum torque output and moderate repetitions. Muscles must be activated quickly to produce maximal power, and the rapid force also correlates with the function of both neural activation and muscle contraction velocities^2,3,52^. Thus, the torque–velocity (T–V) and power–velocity (P–V) relationships are frequently used to evaluate the contractile and functional consequences of muscle fatigue^52,53,55^.

Muscular power is generated and can be measured during dynamic contractions associated with an applied force, regardless of speed^55,56^. However, muscles must be activated quickly to produce maximal power, and maximal power development usually occurs at velocities where force is moderate^3,24,55^. Indeed, maximal power output occurred at the point representing one-half of the projected maximal velocity and one-half of the projected maximal torque^3,24,55^. Thus, 50% IPT was the acceptable highest resistance for subjects to produce the maximal power output during each contraction in the fatigue protocol of the IME test^3,14,57^, and muscle fatigue was defined as a time-dependent loss in maximal power output. Different from fatigue protocols used by previous studies^15,30–33,58^, the duration or number of repetitive contractions of the IME protocol in the present study was the total output until the subject could not carry on and voluntarily gave up. The limit of a subject’s endurance during the IME test, that is, the point at which the subject is unwilling or unable to continue repetitive contractions, has been referred to as the point of ‘exhaustion’, ‘the limit of tolerance’, or the point of ‘fatigue’^4,5^. I n this study, two of the same mechanical manifestations of muscle fatigue found in previous studies were observed: one was a significant difference in power output between the first five repetitions and the last five repetitions, and the other was a progressive decrease of power output as the number of repetitions increased^1,14,39,59^. T herefore, by keeping all of the variables consistent except for time, muscular endurance can be measured by considering the amount of work produced or the activity duration via the recommended IME testing protocol in this study.

 The electrical activity of muscle fibers can be characterized by sEMG and used to indirectly assess muscle fatigue caused by the prolongation of muscle contractions over time^17,60^. Traditional parameters such as amplitude-based parameters or spectral parameters based on FT may be questionable for mapping muscle fatigue due to the non-stationarity of the sEMG signal during dynamic contractions^12,13,30,46^. Similar to previous studies^28,30,45^, the results of this study also showed that the use of traditional time-domain indices or spectrum indices alone were not sensitive enough to determine muscle fatigue due to their poor association with power output, and these indices as single parameter predictors accounted for a low percentage of the performance variance of changes in power output. Studies addressing the non-stationarity of the sEMG signal confirmed that the CWT had the best accuracy and estimation capacity on a simulated data test and better accuracy for mapping changes in sEMG signals recorded during dynamic contractions when compared with other time–frequency distributions, and the indices of fatigue based on the CWT were instantaneous frequency parameters^12,13,32^. Using the CWT, which enables variable window sizes in analyzing different frequency components within a signal, a shift toward lower frequencies and, thus, a decrease in the IMNF and IMDF over the fatiguing dynamic contractions were observed^32^. Practically, these two indices provided greater accuracy to map losses in power output than the traditional sEMG-based parameters. In addition, some common behavior was identified, such as that the RMS was more sensitive than MAV, MDF was more sensitive than MNF^17,46,51^, and IMDF was preferable to IMNF as it had higher sensitivity and lower estimation error^17,51,61,62^.

 For linear versus non-linear mapping of power loss during dynamic fatiguing contractions, some studies showed that a non-linear model based on a learning procedure provided more accurate tracking of fatigue using sEMG variables during isometric, isokinetic, and random elbow contractions^12,34^. However, another study showed that the linear and non-linear approaches were equally valid to estimate changes in power loss during a fatiguing repetitive leg extension exercise^12,31^. In the present study, non-linear techniques were developed using an MLPNN to combine different time and spectral features of the sEMG signal into a fatigue index representing the estimated changes in power output^12,31,34,35^. The results showed that the estimation errors were smaller when using non-linear mapping of power loss during dynamic contractions compared to linear mapping, as it showed higher SNR and correlation coefficients between the actual and estimated power output in three muscle groups of lower limbs. Therefore, non-linear mapping is a more powerful approach for training the network with data from all subjects, and using just one neural network can provide a more general technique to track changes in power loss for different subjects^12,15,51^. Differences in accuracy may be associated with different fatigue protocols, muscle groups, and muscle actions, but these differences remain unclear and of potential interest for further studies. Furthermore, it should be noted that non-linear techniques provided more accurate mapping of power loss but required more computational time.

 Isotonic and isokinetic movements are two dynamic methods used to assess muscle power and improve muscle strength, and their respective superiority has been demonstrated^27,57^. Isokinetic testing controls the angular velocity and allows the force or torque to vary as muscle output changes, and it tends to “accommodate” the patient’s effort and is mainly used to measure the muscular power under constant velocity during rehabilitation^28,53,63^. Isotonic testing is considered to be more relevant to normal voluntary contractions, as the load is held constant but the velocity can vary^27,28^. Thus, the isotonic testing protocol and corresponding database presented herein should have wide application in the evaluation of sports science, rehabilitation medicine, and human-factor engineering. For instance, it is more important to maximize the neural drive than to increase absolute force levels during the early stages of rehabilitation, so clinicians have been recommended to incorporate early isotonic training and evaluation, as those contractions resulted in greater motor unit activation per unit of work performed^2,57^. In addition, the isotonic testing protocol is beneficial for developing and evaluating training programs that effectively enhance maximal power production, since sports activities involve the acceleration and deceleration of the lower extremities^22,23^. Moreover, dynamic contractions are more closely related to day-to-day activities and are psychologically more demanding since they require both movement and postural control^36^. The debilitating effects of increased physiological strain on endurance performance are accompanied by debilitating effects of increased perceived fatigability^19,20^. When evaluating physical ability or functionality related to work activities, an IME test might offer more convincing and accurate results that provide further insights into the relationships between human performance and muscle endurance required in real-world activities^19,40^.

To develop electrophysiological evaluation methods of muscle fatigue in IME tests, linear mapping alone and a combination of sEMG variables with non-linear mapping of power loss were compared, indicating that the non-linear techniques (e.g., MLPNN) might have an advantage in analyzing non-linear dynamic systems (e.g., the IME testing protocol). Practically, the linear technique may be preferable for mapping power changes based on sEMG variables due to the lower computational time required by the linear approach^12,17,60^. Fatiguability was examined by assessing the subject’s endurance time until they experienced fatigue and decided to stop, and the protocol may better fit the views in which they considered fatigue as a safety mechanism aimed at preventing overuse injuries in previous studies^6,7,9^. However, there may be different mechanisms behind fatigue development and exhaustion in the IME testing protocol compared with previous dynamic fatiguing protocols using fixed sets, repetitions, and rest times^15,30–33,39^. This may partially explain the low correlations between power loss and the sEMG-based parameters, but further research is needed. Moreover, the sensitivity of other proposed fatigue indices, such as Dimitrov’s spectral fatigue index (F_insmk_), wavelet spectral parameters, the ratios of EMG power content in the high- and low-frequency ranges (F_ihlrx_), and other non-linear parameters^12,16,17,51,64^, was not investigated. Further studies can be conducted to determine more effective electrophysiological evaluation methods of neuromuscular function while considering the parameters in the IME testing protocol. 

## Conclusion 

Muscular endurance required in real-world activities can be determined by measuring the amount of work produced or the activity duration via the recommended IME testing protocol under a psychophysiological termination criterion. Furthermore, non-linear mapping techniques provide more powerful mapping of power loss compared to linear mapping techniques for the IME testing protocol.

## Data availability

The datasets used and/or analyzed during the current study are available from the corresponding author on reasonable request.

## Abbreviations

ANOVA: Analysis of variance
AV: Actual value
BF: Biceps femoris
CV: Coefficient of variation
CWT: Continuous wavelet transform
EV: Estimated value
F_ihlrx_: High and low frequency ranges
F_insmk_: Dimitrov’s spectral fatigue index
FT: Fourier transform
GM: Gluteus maximus
HT: Hilbert transform
IMDF: Instantaneous median frequency
IME: Isotonic muscular endurance
IMNF: Instantaneous mean frequency
IPT: Isometric peak torque
LG: Lateral gastrocnemius
MAV: Mean absolute value
MDF: Median frequency
MG: Medial gastrocnemius
MLPNN: Multilayer perceptron neural network
MNF: Mean frequency
MVC: Maximum voluntary isometric contraction
r: Pearson’s correlation coefficient
R^2^: Coefficient of determination
RF: Rectus femoris
RMS: Root mean square
sEMG: Surface electromyography
SNR: Signal-to-noise ratio
SSC: Slope sign changes
VL: Vastus lateralis
VM: Vastus medialis
WL: Wavelength
ZC: Zero crossings

## References

[1] Murillo-Escobar J, Jaramillo-Munera YE, Orrego-Metaute DA, Delgado-Trejos E, Cuesta-Frau D (2020). Muscle fatigue analysis during dynamic contractions based on biomechanical features and Permutation Entropy. Math. Biosci. Eng. MBE.

[2] Akagi R, Hinks A, Davidson B, Power GA (2020). Differential contributions of fatigue-induced strength loss and slowing of angular velocity to power loss following repeated maximal shortening contractions. Physiol. Rep..

[3] Stauber WT, Barill ER, Stauber RE, Miller GR (2000). Isotonic dynamometry for the assessment of power and fatigue in the knee extensor muscles of females. Clin. Physiol. (Oxford, Engl.).

[4] Burnley M, Jones AM (2018). Power-duration relationship: Physiology, fatigue, and the limits of human performance. Eur. J. Sport Sci..

[5] James A, Green S (2012). A phenomenological model of muscle fatigue and the power-endurance relationship. J. Appl. Physiol. (Bethesda, Md.: 1985).

[6] Abbiss CR, Laursen PB (2005). Models to explain fatigue during prolonged endurance cycling. Sports Med. (Auckland, N.Z.).

[7] Ament W, Verkerke GJ (2009). Exercise and fatigue. Sports Med (Auckland, N.Z.).

[8] Van Cutsem J (2017). The effects of mental fatigue on physical performance: a systematic review. Sports Med. (Auckland, N.Z.).

[9] Vøllestad NK (1997). Measurement of human muscle fatigue. J. Neurosci. Methods.

[10] Potvin JR, Fuglevand AJ (2017). A motor unit-based model of muscle fatigue. PLoS Comput. Biol..

[11] Gacesa JZ, Klasnja AV, Grujic NG (2013). Changes in strength, endurance, and fatigue during a resistance-training program for the triceps brachii muscle. J. Athl. Train.

[12] Gonzalez-Izal M, Malanda A, Gorostiaga E, Izquierdo M (2012). Electromyographic models to assess muscle fatigue. J. Electromyogr. Kinesiol. Off. J. Int. Soc. Electrophysiol. Kinesiol..

[13] Shair EF, Ahmad SA, Marhaban MH, Mohd Tamrin SB, Abdullah AR (2017). EMG processing based measures of fatigue assessment during manual lifting. Biomed Res Int.

[14] Wan JJ, Qin Z, Wang PY, Sun Y, Liu X (2017). Muscle fatigue: general understanding and treatment. Exp. Mol. Med..

[15] Gonzalez-Izal M, Falla D, Izquierdo M, Farina D (2010). Predicting force loss during dynamic fatiguing exercises from non-linear mapping of features of the surface electromyogram. J. Neurosci. Methods.

[16] Kim H, Lee J, Kim J (2018). Electromyography-signal-based muscle fatigue assessment for knee rehabilitation monitoring systems. Biomed. Eng. Lett..

[17] Rampichini S, Vieira TM, Castiglioni P, Merati G (2020). Complexity analysis of surface electromyography for assessing the myoelectric manifestation of muscle fatigue: a review. Entropy.

[18] Cifrek M, Medved V, Tonkovic S, Ostojic S (2009). Surface EMG based muscle fatigue evaluation in biomechanics. Clin. Biomech. (Bristol, Avon).

[19] Enoka RM, Duchateau J (2016). Translating fatigue to human performance. Med. Sci. Sports Exerc..

[20] Venhorst A, Micklewright DP, Noakes TD (2018). The psychophysiological regulation of pacing behaviour and performance fatigability during long-distance running with locomotor muscle fatigue and exercise-induced muscle damage in highly trained runners. Sports Med. Open.

[21] Kankaanpää M, Taimela S, Webber CL, Airaksinen O, Hänninen O (1997). Lumbar paraspinal muscle fatigability in repetitive isoinertial loading: EMG spectral indices, Borg scale and endurance time. Eur. J. Appl. Physiol..

[22] Cormie P, McGuigan MR, Newton RU (2011). Developing maximal neuromuscular power: Part 1–biological basis of maximal power production. Sports Med. (Auckland, N.Z.).

[23] Cormie P, McGuigan MR, Newton RU (2011). Developing maximal neuromuscular power: part 2 - training considerations for improving maximal power production. Sports Med. (Auckland, N.Z.).

[24] Alcazar J, Csapo R, Ara I, Alegre LM (2019). On the shape of the force-velocity relationship in skeletal muscles: the linear, the hyperbolic, and the double-hyperbolic. Front Physiol..

[25] Webber SC, Porter MM (2010). Reliability of ankle isometric, isotonic, and isokinetic strength and power testing in older women. Phys. Ther..

[26] Frykholm E (2019). Inter-day test-retest reliability and feasibility of isokinetic, isometric, and isotonic measurements to assess quadriceps endurance in people with chronic obstructive pulmonary disease: a multicenter study. Chron. Respir. Dis..

[27] Cairns SP, Knicker AJ, Thompson MW, Sjøgaard G (2005). Evaluation of models used to study neuromuscular fatigue. Exerc. Sport Sci. Rev..

[28] Park JH, Chung SW, Lee SJ, Lee JW, Oh KS (2020). Evaluation of the electromyographic amplitude-to-work ratio in the infraspinatus muscle during external shoulder rotation exercises: a comparison of concentric isotonic and isokinetic exercises. Orthop. J. Sports Med..

[29] Pua YH, Ho JY, Chan SA, Khoo SJ, Chong HC (2017). Associations of isokinetic and isotonic knee strength with knee function and activity level after anterior cruciate ligament reconstruction: a prospective cohort study. Knee.

[30] Gonzalez-Izal M (2010). EMG spectral indices and muscle power fatigue during dynamic contractions. J. Electromyogr. Kinesiol. Off. J. Int. Soc. Electrophysiol. Kinesiol..

[31] Gonzalez-Izal M (2010). Linear vs. non-linear mapping of peak power using surface EMG features during dynamic fatiguing contractions. J. Biomech..

[32] Gonzalez-Izal M (2010). sEMG wavelet-based indices predicts muscle power loss during dynamic contractions. J. Electromyogr. Kinesiol. Off. J. Int. Soc. Electrophysiol. Kinesiol..

[33] Longpre HS, Potvin JR, Maly MR (2013). Biomechanical changes at the knee after lower limb fatigue in healthy young women. Clin. Biomech. (Bristol, Avon).

[34] MacIsaac DT, Parker PA, Englehart KB, Rogers DR (2006). Fatigue estimation with a multivariable myoelectric mapping function. IEEE Trans. Biomed. Eng..

[35] Ma F, Song F, Liu Y, Niu J (2020). sEMG-based neural network prediction model selection of gesture fatigue and dataset optimization. Comput. Intell. Neurosci..

[36] Hussain J, Sundaraj K, Subramaniam ID (2020). Cognitive stress changes the attributes of the three heads of the triceps brachii during muscle fatigue. PLoS ONE.

[37] Ahmad I, Kim JY (2018). Assessment of whole body and local muscle fatigue using electromyography and a perceived exertion scale for squat lifting. Int. J. Environ. Res. Publ. Health.

[38] Cowley JC, Gates DH (2017). Proximal and distal muscle fatigue differentially affect movement coordination. PLoS ONE.

[39] Lee A (2017). Sex differences in neuromuscular function after repeated eccentric contractions of the knee extensor muscles. Eur J Appl Physiol.

[40] Aleksandrov AA, Knyazeva VM, Stankevich LN, Dmitrieva ES, Shestakova AN (2016). Mismatch Negativity Affects Muscle Fatigue during Repeated Contraction Trials of Different Durations. Front Physiol.

[41] Merletti R, Muceli S (2019). Tutorial. Surface EMG detection in space and time: best practices. J. Electromyogr. Kinesiol. Off. J. Int. Soc. Electrophysiol. Kinesiol..

[42] De Luca CJ, Kuznetsov M, Gilmore LD, Roy SH (2012). Inter-electrode spacing of surface EMG sensors: reduction of crosstalk contamination during voluntary contractions. J. Biomech..

[43] Afsharipour B, Soedirdjo S, Merletti R (2019). Two-dimensional surface EMG: the effects of electrode size, interelectrode distance and image truncation. Biomed. Signal Process. Control.

[44] Melaku, A., Kumar, D. K. & Bradley, A. In *2001 Conference Proceedings of the 23rd Annual International Conference of the IEEE Engineering in Medicine and Biology Society.* Vol. 1082, pp. 1082–1085 (2001)

[45] Gerdle B, Larsson B, Karlsson S (2000). Criterion validation of surface EMG variables as fatigue indicators using peak torque: a study of repetitive maximum isokinetic knee extensions. J. Electromyogr. Kinesiol. Off. J. Int. Soc. Electrophysiol. Kinesiol..

[46] Bilodeau M, Schindler-Ivens S, Williams DM, Chandran R, Sharma SS (2003). EMG frequency content changes with increasing force and during fatigue in the quadriceps femoris muscle of men and women. J. Electromyogr. Kinesiol. Off. J. Int. Soc. Electrophysiol. Kinesiol..

[47] Kilby, J. & Hosseini, H. G. Extracting effective features of SEMG using continuous wavelet transform. In *Conference proceedings: Annual International Conference of the IEEE Engineering in Medicine and Biology Society. IEEE Engineering in Medicine and biology Society. Annual Conference* **1**, 1704–1707, 10.1109/iembs.2006.260064 (2006). 10.1109/IEMBS.2006.260064 17946475

[48] Srhoj-Egekher V, Cifrek M, Medved V (2011). The application of Hilbert-Huang transform in the analysis of muscle fatigue during cyclic dynamic contractions. Med. Biol. Eng. Compu..

[49] Xie H, Wang Z (2006). Mean frequency derived via Hilbert-Huang transform with application to fatigue EMG signal analysis. Comput. Methods Progr. Biomed..

[50] Hudgins B, Parker P, Scott RN (1993). A new strategy for multifunction myoelectric control. IEEE Trans. Biomed. Eng..

[51] Chowdhury RH (2013). Surface electromyography signal processing and classification techniques. Sensors (Basel).

[52] Kruger RL (2019). Fatigue and recovery measured with dynamic properties versus isometric force: effects of exercise intensity. J. Exp. Biol..

[53] Wang X, Tao X, So RCH (2017). A bio-mechanical model for elbow isokinetic and isotonic flexions. Sci Rep.

[54] Cheng AJ, Rice CL (2005). Fatigue and recovery of power and isometric torque following isotonic knee extensions. J. Appl. Physiol. (Bethesda, Md.: 1985).

[55] Devrome AN, MacIntosh BR (2018). Force-velocity relationship during isometric and isotonic fatiguing contractions. J. Appl. Physiol. (Bethesda, Md.: 1985).

[56] Mallor F, Leon T, Gaston M, Izquierdo M (2010). Changes in power curve shapes as an indicator of fatigue during dynamic contractions. J. Biomech..

[57] Schmitz RJ, Westwood KC (2001). Knee extensor electromyographic activity-to-work ratio is greater with isotonic than isokinetic contractions. J. Athl. Train.

[58] Walker S, Peltonen J, Ahtiainen JP, Avela J, Hakkinen K (2009). Neuromuscular fatigue induced by an isotonic heavy-resistance loading protocol in knee extensors. J. Sports Sci..

[59] Enoka RM, Duchateau J (2008). Muscle fatigue: what, why and how it influences muscle function. J. Physiol..

[60] Bueno, D. R., Lizano, J. M. & Montano, L. Muscular fatigue detection using sEMG in dynamic contractions. In *Conference proceedings: Annual International Conference of the IEEE Engineering in Medicine and Biology Society. IEEE Engineering in Medicine and Biology Society. Annual Conference* **2015**, 494–497, doi:10.1109/embc.2015.7318407 (2015). 10.1109/EMBC.2015.7318407 26736307

[61] Bonato P, Roy SH, Knaflitz M, De Luca CJ (2001). Time-frequency parameters of the surface myoelectric signal for assessing muscle fatigue during cyclic dynamic contractions. IEEE Trans. Biomed. Eng..

[62] Karlsson S, Yu J, Akay M (2000). Time-frequency analysis of myoelectric signals during dynamic contractions: a comparative study. IEEE Trans. Biomed. Eng..

[63] Purkayastha S, Cramer JT, Trowbridge CA, Fincher AL, Marek SM (2006). Surface electromyographic amplitude-to-work ratios during isokinetic and isotonic muscle actions. J. Athl. Train.

[64] Kim J, Park S, Ahn S, Kim YJIJOPE (2012). Manufacturing. A novel approach of defining fatigue indices with sEMG power during isotonic contractions. Int. J. Precis. Eng. Manuf..

